# Design of Environmentally Friendly Ca-Alginate Beads for Self-Healing Cement-Based Materials

**DOI:** 10.3390/ma15175844

**Published:** 2022-08-24

**Authors:** Xiaohang Zhang, Yonggang Ding

**Affiliations:** School of Civil Engineering and Architecture, Henan University of Technology, Zhengzhou 450001, China

**Keywords:** ca-alginate, protonation theory, cement-based materials, internal curing, self-healing

## Abstract

Ca-alginate beads have strong hygroscopicity, which have been used for the self-healing and internal curing of cement-based materials. However, ca-alginate beads in cement will chelate with calcium ions, which decreases the swelling rate of ca-alginate beads in the healing environment and is detrimental to the self-healing of cement-based materials. In this paper, the mechanism and steps for preparing ca-alginate beads with a lower ability to chelate with calcium ions were proposed based on protonation theory. In addition, the molecular structure and the swelling rates in cement filtrate and healing environment of ca-alginate beads prepared by the proposed method were characterized. The results showed that the ca-alginate beads prepared by the proposed method had higher molecular density and a lower ability to chelate with calcium ions. The swelling rate in the healing environment is not decreased. Furthermore, the equilibrium swelling rate in cement filtrate can satisfy the need for internal curing of cement-based materials.

## 1. Introduction

Cement is one of the most important building materials. However, the manufacturing process of cement releases a large amount of carbon dioxide, which accounts for 5–7% of carbon dioxide emissions [1,2]. The carbon dioxide emissions and the consumption of cement can be reduced by extending the service life of the structure. However, cement-based materials are brittle and prone to cracks [3]. The generation of cracks accelerates the intrusion of harmful ions and, thus, reduces the service life of cement-based materials [4]. Therefore, it has become an urgent issue for governments around the world to extend the service life of the structure.

Abrams found that cracks in cement-based materials could self-healing in 1925. The basic principle is that the unhydrated cement particles in the cement matrix undergo secondary hydration under a certain humidity to form calcium silicate hydrate and calcium carbonate. However, only the small cracks of 30–50 μm can be healed in cement-based materials [5].

Superabsorbent polymer (SAP) has been used to strengthen the self-healing ability of cement-based materials [6]. In order to ensure self-healing efficiency, SAP equal to 1% of the cement quality should be added to the cement [7]. The cracks smaller than 50 μm in cement-based materials mixed with SAP are completely healed, and the cracks smaller than 150 μm are partially healed [5]. In addition, SAP is also used for the internal curing of cement-based materials [8,9,10]. The amount of entrained water required for internal curing is 0.18 times the water–cement ratio [8]. Furthermore, SAP can absorb water dozens of times its weight during mixing, which can usually meet the needs of internal curing. However, the excess water is entrained due to the high absorbent ability, which forms a large number of large pores in the cement-based materials after release. The formation of large pores will decrease the strength of cement-based materials [7].

Sodium alginate is a water-soluble anion polysaccharide in brown algae. It has excellent water absorption and moisture retention, which is also environmentally friendly and widely available. Primarily, it is a polysaccharide polymer ((C_6_H_7_O_6_Na)_n_) composed of 1–4 poly β-D-mannuronic acid (M) and α-L-guluronic acid (G) monomers. Here, ca-alginate beads are formed by the combination of the carboxyl group on sodium alginate and calcium ions by an ionic bond and chelation bond. The ca-alginate beads have a low swelling rate and stable structure. The strength of cement-based materials with 1 wt% ca-alginate beads is 13% higher than that of cement-based materials mixed with 1 wt% SAP under the water-cement ratio of 0.5 when tested at 28 days [11]. In addition, ca-alginate beads have strong hygroscopicity under the relative humidity of the atmosphere, which can be used for self-healing of cement-based materials in the region where rain is scarce [11]. The ca-alginate beads can also act as a precursor of calcium carbonate crystals to promote cement hydration [12,13,14,15]. Therefore, the ca-alginate bead is a promising material for cement-based materials.

Enough water needs to be entrained when ca-alginate beads are used for the internal curing of cement-based materials as an absorbent material, which means that the amount of entrained water is 0.18 times the water–cement ratio. In addition, the swelling rate of ca-alginate beads in the healing environment should be much greater than that in the cement paste to ensure the efficiency of the self-healing [6]. However, ca-alginate beads in the cement filtrate chelate with calcium ions, which increases the crosslinking density of ca-alginate beads [11]. When the cement-based materials cracks, the swelling rate of ca-alginate beads will decrease due to the increase in crosslinking density, which is not conducive to self-healing. Therefore, the ability to chelate with calcium ions in the cement filtrate needs to be reduced for the ca-alginate beads applied for the self-healing of cement-based materials.

The ca-alginate bead is a kind of hydrogel with a controllable three-dimensional structure [16]. The ability to chelate with calcium ions can be decreased by increasing the concentration of sodium alginate [17]. The concentration of sodium alginate solution has a limited value due to the viscosity of the sodium alginate solution. The maximum concentration of sodium alginate solution is 8% in the study by Stewart [17]. However, the existing preparation methods all need to prepare a sodium alginate solution first, then disperse the sodium alginate solution into beads and expose them to the calcium salt solution to chelate with calcium ions to form ca-alginate beads [16,17]. Therefore, it is difficult to increase the concentration of sodium alginate solution using the existing methods.

Sodium alginate has a reversible pH sensitivity. Part of –COO– in sodium alginate will be protonated and become –COOH in an acidic solution [18]. On the one hand, this will reduce the hydrophilicity of sodium alginate but, on the other hand, hydrogen bonds can be formed between –COOH, which makes the protonated segments become insoluble in water and precipitate out of the solution [18]. Additionally, Cao makes the sodium alginate solution self-assemble to form sodium alginate micelles in acidic solution based on protonation theory, and then makes the micelles exchange and chelate with calcium ions to form ca-alginate beads.

In this paper, the mechanism and steps of the preparation method for ca-alginate beads with a higher concentration of sodium alginate were proposed based on protonation theory. The molecular structure, content of the chelation bond, morphology, and pore structure of the ca-alginate beads prepared by this method were characterized. In addition, its potential for cement-based materials was discussed based on its swelling behavior in the cement filtrate and the healing environment.

## 2. Materials and Methods

### 2.1. Materials

#### 2.1.1. Cement

Portland cement with 42.5 strength grade (P∙I 42.5) produced by the China Building Materials Institute was used. The chemical composition of the cement was identified by the X-ray fluorescence test. The cement mainly consists of CaO, SiO_2_, SO_3_, Fe_2_O_3_, Al_2_O_3_, MgO, and K_2_O. The content is 64.20 wt%, 22.37 wt%, 2.39 wt%, 3.18 wt%, 4.07 wt%, 2.06 wt%, and 0.45 wt%, respectively.

#### 2.1.2. Alginate

The sodium alginate was provided by Macklin, Shanghai, China. The ratio between mannuronic and guluronic acid was about 1.5. The particle size of the sodium alginate was below 200 μm. The viscosity of a 10% solution was 200 ± 20 mPa·s at 20 °C.

#### 2.1.3. Chemicals

The deionized water and anhydrous ethanol were provided by Macklin, China. The calcium chloride with a purity of 99.99% was provided by Macklin, China.

### 2.2. The Preparation Method of Ca-Alginate Beads

#### 2.2.1. Preparation Mechanism and Steps of Ca-Alginate Beads with a Higher Concentration of Sodium Alginate

**Figure 1:**
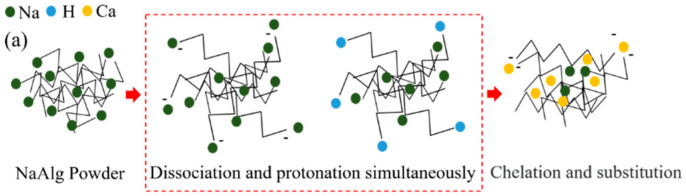

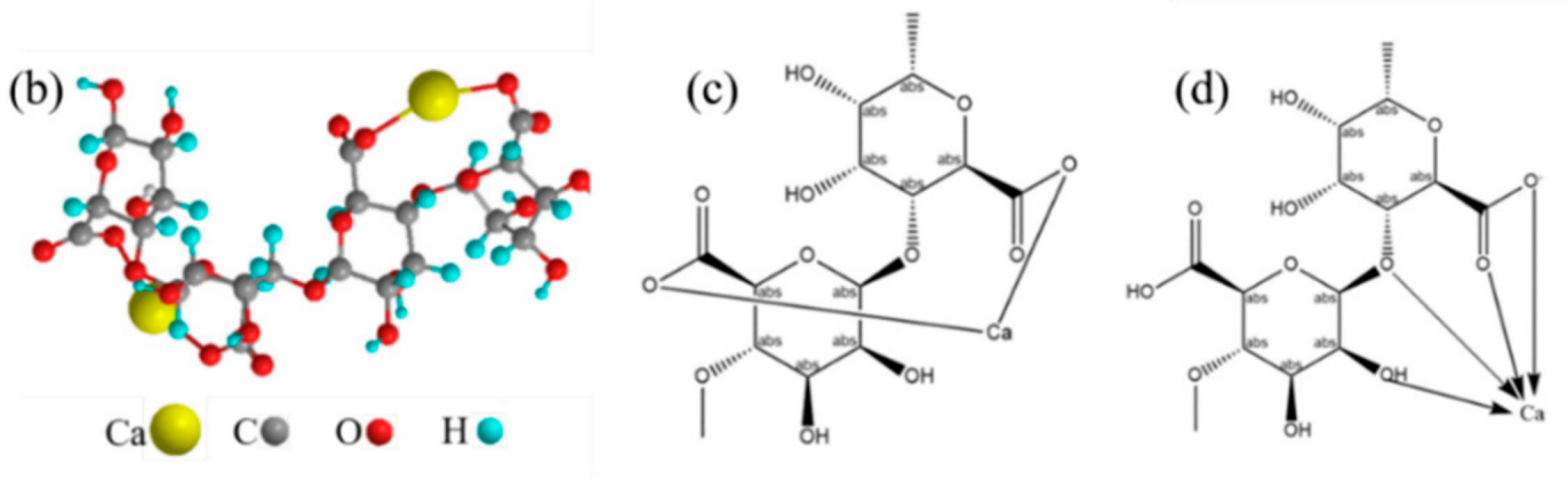
The preparation schematic diagram of PCA and the molecular structure of ca-alginate: (**a**) preparation schematic diagram of PCA, (**b**) the molecular chain of ca-alginate, (**c**) ionic bond, and (**d**) chelation.

Sodium alginate powder is directly protonated in an acid solution to obtain calcium alginate beads (PCA) with a higher concentration of sodium alginate. The preparation mechanism of ca-alginate beads (PCA) is based on the protonation theory (shown in Figure 1a). The sodium alginate powder will be dissolved and protonated at the same time when it is put into an acid solution, whose PH value is lower than the pKa value of sodium alginate. The molecular chain of the outer layer of sodium alginate stretches gradually, and the –COONa in the molecular chain dissociates into –COO– and Na^+^. Part of –COO– in sodium alginate is protonated and becomes -COOH. Additionally, hydrogen bonds can also be formed between –COOH, which reduces the surface charge of sodium alginate and, thus, decreases the charge repulsion between the powders [18]. The hydrophobic shell is formed in the surface layer of sodium alginate. The protonated sodium alginate powder will precipitate out of the solution when the protonation reaches a certain degree. Then, the protonated sodium alginate powder is mixed with calcium salt and the pH value of the acid solution is increased at the same time. In the process, ionic bonds (Figure 1c) and chelate bonds (Figure 1d) are formed between Ca^2+^ and alginate molecules. In the end, the pH of the solution is adjusted to about 7 to remove H^+^ and, thus, ca-alginate beads are formed.

Sodium alginate is protonated and dispersed when the pH value is less than the pKa value (≈3). The disperse phenomenon of sodium alginate powder in acid solutions with different pH values is different (the phenomenon shown in Table 1). The sodium alginate powder disperses better in an acid solution with a pH value of 0.01, but it disperses poorly and undergoes agglomeration in an acid solution with a higher pH value. The main reason for the agglomeration of sodium alginate in acid solution is the insufficient hydrogen ion content in the acid solution, which causes the molecular chain on the surface of sodium alginate to be protonated insufficiently and to agglomerate due to a lack of charge repulsion. The larger size of ca-alginate beads will be obtained due to the agglomeration. The large size is detrimental to the strength of cement-based materials [19]. In order to obtain the smaller size of ca-alginate beads, sodium alginate powder need to undergo better dispersion and less agglomeration. Strong acid with a pH value of about 0.01 was selected during preparation to obtain good dispersion. The sodium alginate powder should avoid contact with water before adding to the acid solution to avoid agglomeration and poor dispersion. Therefore, the preparation container was wetted with strong acid first.

**Figure 2:**
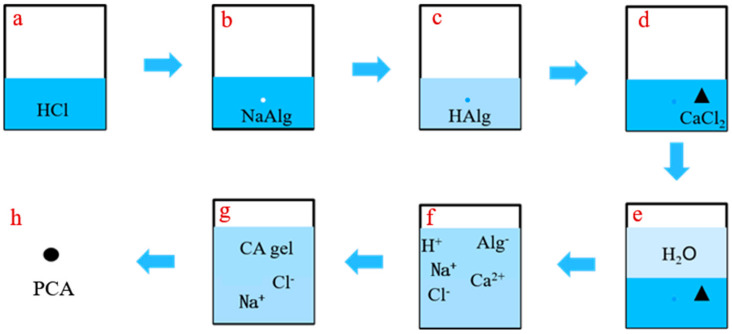
Ion changes in the preparation process of PCA.

**Figure 3:**
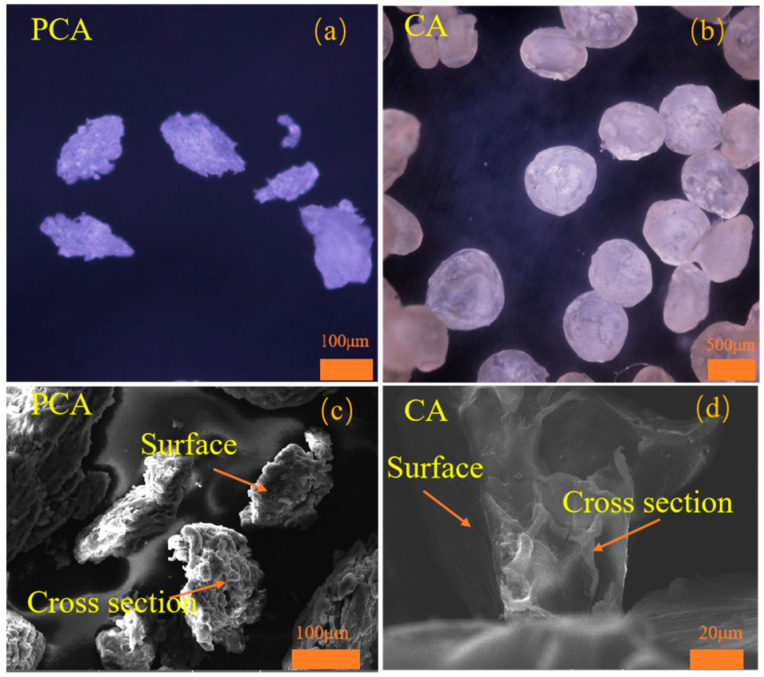
The morphology of PCA and CA: (**a**,**c**) the morphologies of PCA, (**b**,**d**) the morphologies of CA.

**Figure 4:**
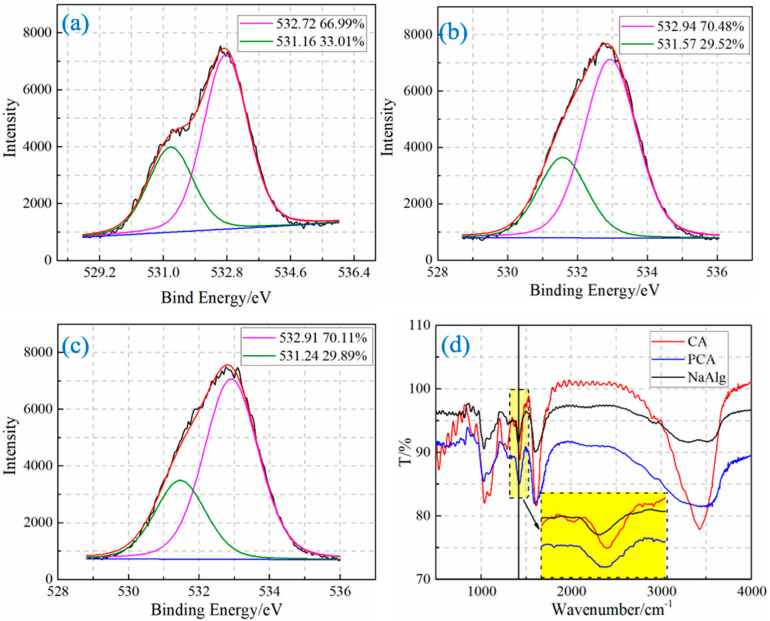
The results of XPS and FTIR: (**a**) the peaks splitting and fitting of O1s high-definition spectrogram of sodium alginate, (**b**) the peaks splitting and fitting of O1s high-definition spectrogram of CA, (**c**) the peaks splitting and fitting of O1s high-definition spectrogram of PCA, (**d**) the FTIRs of PCA, CA, and sodium alginate. Note: In Figure 4a–c, the black line represents the original data, the red line represents the curve after fitting, and the pink and green lines are the curves after peaking according to the fitted curve.

**Figure 5:**
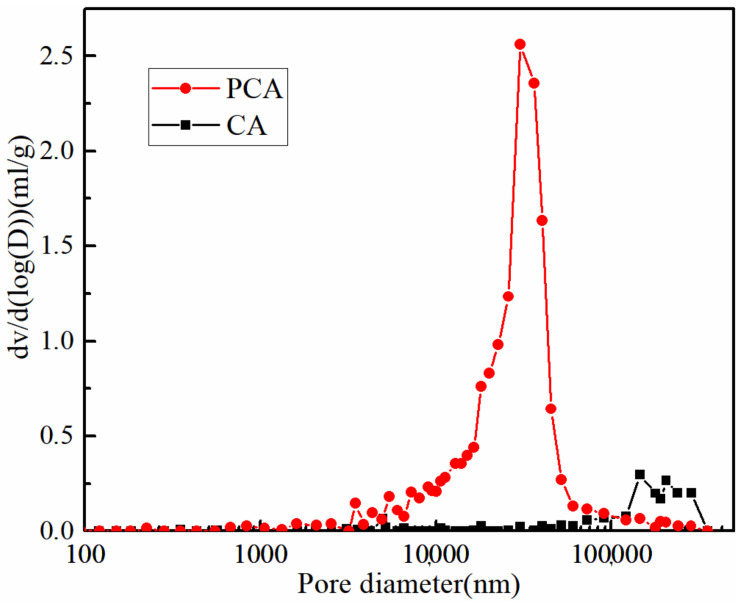
Pore diameter distributions of two kinds of ca-alginate beads.

The preparation steps of ca-alginate beads (PCA) were determined according to the preparation principle and the disperse phenomenon of sodium alginate in acid solutions with different pH values, as follows:

(1) Wetting the wall of the container with concentrated hydrochloric acid with a pH value of less than 0.01, and then adding 50 mL of the concentrated hydrochloric acid to the container;
(2) Turning on the magnetic stirrer and adding 50 g of sodium alginate powder to the acid while stirring slowly, so that the sodium alginate powders were protonated and dispersed better. The ion changes are shown in Figure 2b,c;
(3) Adding 50 g calcium chloride powder and stirring evenly, then adding deionized water to ensure the total volume of the solution in the reaction container was 2.25 L and the concentration of calcium chloride was 0.2 mol/L. The ion changes are shown in Figure 2d–f;
(4) Adjusting the pH value of the solution to about 7 with sodium hydroxide and stirring for 24 h to fully react the molecular chain of sodium alginate and calcium ions to form chelate bonds and ionic bonds. The ion changes are shown in Figure 2f,g;
(5) Washing and filtering the prepared ca-alginate beads with deionized water at least three times to remove excess ions;
(6) Putting the washed ca-alginate beads into a drying oven at 40 °C until the mass was constant. The dried ca-alginate beads (PCA) were obtained.

#### 2.2.2. Preparation Method of Ca-Alginate Beads with a Lower Concentration of Sodium Alginate

The dropwise addition method was used to prepare ca-alginate beads (CA) with a lower concentration of sodium alginate [17]. The concentration of sodium alginate and the concentration of calcium chloride in this method were 2 wt% and 0.2 mol/L, respectively. The reaction system was stirred for 24 h to react fully between the molecular chain of sodium alginate and calcium ions to form chelate bonds and ionic bonds after the sodium alginate beads were dropped into the solution of calcium chloride with a syringe. After that, the ca-alginate beads were obtained. Then, the ca-alginate beads were washed and filtered with deionized water at least three times to remove excess ions. In the end, the washed ca-alginate beads were put into a drying oven at 40 °C until the mass was constant. The dried ca-alginate beads (CA) were obtained.

### 2.3. Methods

#### 2.3.1. Molecular Structures

The Nicolet is 5003190721 Fourier infrared spectrometer (FTIR-650) with a scanning resolution of 0.482 cm^−1^, which was used to characterize the molecular structure of ca-alginate beads. The scanning range was 500 cm^−1^–4000 cm^−1^.

Here, X-ray photoelectron spectroscopy (XPS) was used to analyze the combination form of calcium ions and carboxyl groups. A 250Xi ESCLAB (Shanghai, China) photoelectron spectrometer with Al K_α_ rays was used. The pass energy of 100 eV and 20 eV were used for wide-range scanning spectra and narrow-range scanning spectra (high resolution) of C1_s_ and O1_s_, respectively. The XPS system software and instrument standard parameters were used for spectrum analysis and element composition quantification. The Origin mathematics software was used for high-resolution spectra to subtract linear background and split peaks with 80% Gaussian −20% Lorentzian mixed function. The C1_s_ binding energy of saturated hydrocarbons, such as diffusion pump oil and other pollutants (284.8 eV), was used to correct the binding energy [20,21,22,23].

#### 2.3.2. Morphology and Size

A DSX500 ultra-depth-of-field optical digital microscope produced by Japan’s OLYMPUS company (Shinjuku, Japan) was used to photograph the morphology of two kinds of ca-alginates beads. The BT-2001 laser particle size analyzer (Liaoning, China) was used to test the size distribution and average size of the two kinds of ca-alginates beads.

The morphology images were also obtained using a scanning electron microscope (SEM) produced by the TESCAN company (Brno, Czech Republic), with a VEGA3 tungsten filament at a magnification of 499 times and 1840 times in vacuum conditions at 30 kV. The samples for SEM were vacuum dried and coated with a thin layer of gold on the surface.

#### 2.3.3. Pore Structure

Before the test, the prepared samples were dried in a 50 °C dryer for 24 h. The Auto-Pore IV9500 mercury porosimeter (Norcross, GA, USA, with a pressure range of 0 to 60,000 psi) was used to measure the pore size and pore distribution of the interconnected pores of PCA and CA. The powder dilatometer was selected for the test. Firstly, the drying sample (about 0.05 g) and the dilatometer assembly were weighed. The head of the dilatometer was sealed with resin. Then, the low-pressure and high-pressure tests were carried out successively. After measurement, the dilatometer was taken out and cleaned.

#### 2.3.4. Swelling Kinetic Experiment

The cement filtrate was obtained by mixing 10 g of cement and 100 g of deionized water, stirring with a magnetic mixer for 3 h, and filtering with filter paper [11]. In order to simulate the self-healing environment in cement, the ca-alginate beads were first put into cement filtrate to adsorb and react with calcium ions, then dried and put into deionized water for swelling.

The swelling kinetics was measured with the tea bag method [24]. A small amount of dried ca-alginate beads and pre-moistened tea bags were weighed and recorded as *m*_c_ and *m_f_*, respectively. They were put into cement filtrate or a healing environment. Then, they were taken out at regular intervals and wiped with filter paper to absorb redundant solutions on the surface of the tea bags. Their weight was recorded as *m_t_*. The swelling rate *W*(*t*) at *t* minutes was calculated according to Equation (1). Three samples with identical quality were measured for statistical analysis. The means were used for analyzing the swelling kinetics of ca-alginate beads.

(1) $$W\left(t\right)=\frac{m_{t}-m_{f}-m_{c}}{m_{c}}$$

#### 2.3.5. Statistical Analysis

The statistical analysis was performed on the characterization results of molecular structure, size, and swelling kinetics. The values were reported as the mean ± standard deviation.

## 3. Results and Discussion

### 3.1. Morphology and Size

Figure 3 shows the morphology and cross section of PCA and CA. The magnification was represented by the orange bars. Here, CA is a spherical particle with a smooth surface and looser structure (shown in Figure 3b,d), while PCA is an approximate ellipsoid with a rough surface and denser structure (shown in Figure 3a,c). That is because the sodium alginate used for PCA is not dissolved, and the molecular chain is not stretched. Therefore, the content of molecules per unit volume of PCA is higher and the density of molecules inside PCA is greater [17].

The average size of PCA is 89.90 μm. The average size of CA is 287.64 μm, which is nearly 3.2 times that of PCA (shown in Table 2). The size of PCA is reduced by 197.74 μm, which is significantly smaller than that of CA. Therefore, the size of PCA is more beneficial to cement-based materials [19].

### 3.2. Molecular Structure

#### 3.2.1. Molecular Structure of the Surface of Ca-Alginate Beads

The XPS spectrum was processed to characterize the molecular structures in the surfaces of sodium alginate and ca-alginate beads. Figure 4a shows the high-resolution scan spectra of O1s and the peak splitting results of sodium alginates. Two peaks appear in the peak splitting results of the high-resolution scan spectra of O1s. The lower binding energy peak (531.16 eV) corresponds to the ionic bond between the oxygen of carboxyl and the sodium ions. The carboxyl in sodium alginate is all combined with sodium ions by an ionic bond. The area fraction of oxygen of carboxyl in sodium alginate combined with sodium ions by the ionic bond is 33.01%, which is close to the theoretical calculation value of 33.3% [20,23]. The calculating result indicated that the experimental data and the result obtained by the processing method are reliable.

Two peaks also are found in the high-resolution scan spectra of O1s of CA and PCA (shown in Figure 4b,c). Among them, the area fractions of the lower binding energy peaks (531.57 eV, 531.47 eV) corresponding to the oxygen of carboxyl combined with calcium ions by the ionic bond are 29.52% and 29.89%, respectively. This is because the area fraction of oxygen of carboxyl in sodium alginate combined with sodium ion by ionic bond is the total content of the carboxyl in the ca-alginate beads. The proportion of carboxyl in ca-alginate beads combined with calcium ion by the ionic bond is 88.65% (29.52/33.3) and 89.76% (29.89/33.3), respectively. Therefore, the content of carboxyl forming the chelate bond is 11.35% (1–88.65%) and 10.24% (1–89.76%), respectively. The XPS spectra of two other identical samples of CA and PCA were also obtained for statistical evaluation. The statistical results show that the contents of the chelate bond of CA and PCA are 12.86 ± 1.07% and 11.85 ± 1.15%, respectively.

The chelate bond is also formed in the molecular structure of PCA. Furthermore, the content of the chelate bond of PCA is smaller than that of CA, which proves that the content of the chelate bond in PCA under the same calcium salt concentration is decreased. The decreased chelation is caused by the denser structure of PCA, which hinders the diffusion and crosslinking of calcium ions [17].

#### 3.2.2. The Molecular Structure of Ca-Alginate Beads

The FTIRs of PCA, CA, and sodium alginate (NaAlg) are shown in Figure 4d. The stretching vibration peak of the O-H band (≈3500 cm^−1^) is the characteristic peak of intramolecular bonds, whose peak shape becomes stronger and sharper with the increase in the chelation bonds. However, the characteristic peak of the intermolecular bonds of the O-H (≈3250 cm^−1^) has little change with the increase in the chelation bonds [25]. The peak position (≈3500 cm^−1^) of PCA does not increase significantly compared with that of NaAlg, while that of CA increases significantly. The result proves that the content of the chelation bond of PCA is smaller under the same concentration of calcium salt.

The symmetrical stretching vibration peak (≈1420 cm^−1^) of the carboxyl group shifts to a high wave number when the carboxyl group of sodium alginate is combined with calcium ion by an ionic bond. The more calcium ion content, the more the shift occurs [25]. The symmetrical stretching vibration peak (1422 cm^−1^) of the carboxyl group of PCA has a smaller shift, while the stretching vibration peak (1429 cm^−1^) of the carboxyl group of CA has a larger shift. The change means that fewer ionic bonds are formed by the combination of the carboxyl group and calcium ion in PCA under the same concentration of calcium salt.

The decreased content of the chelation bond and ionic bond proves that calcium ions diffuse slowly inside the PCA.

### 3.3. Pore Structure

The differential distribution curves of the pore structures of PCA and CA are shown in Figure 5. The parameter d*v*/d(log(*D*)) represents the derivative of the pore volume with respect to the log of the hole diameter. The pore size of the connected pores inside the PCA is significantly reduced. In addition, the pore size of PCA is significantly smaller compared with that of CA (shown in Figure 3c,d). Therefore, PCA has a higher molecular density according to the conclusion obtained by Stewart et al., who believe that the pore size inside the high molecular density ca-alginate beads is reduced [17].

### 3.4. Swelling Kinetic in Cement Filtrate and the Healing Environment

#### 3.4.1. Swelling Kinetic in Cement Filtrate

**Figure 6:**
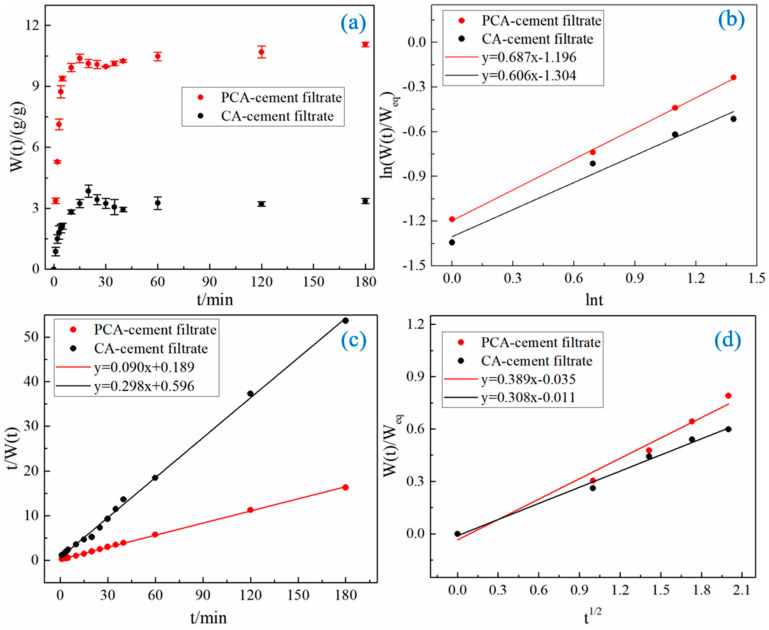
The swelling behaviors of PCA and CA in cement filtrate: (**a**) the swelling rate of two kinds of ca-alginate beads in cement filtrate over time, (**b**) The curve of ln (*W*(*t*)/*W_eq_*) over ln *t*, and (**c**) the curve of *t*/*W*(*t*) over *t*, (**d**) the curve of *W*(*t*)/*W_eq_* over *t*^1/2^.

Figure 6a shows the swelling processes of PCA and CA in the cement filtrate. The two kinds of ca-alginate beads swell rapidly in the first 5 min. Then, the swelling rate increases slowly due to the restriction of the molecular chain. It is interesting that the swelling rate starts to decrease when it reaches the maximum value. The decrease can be explained by other researcher’s conclusions that ca-alginate beads adsorb calcium ions in cement filtrate, which leads to the decrease in crosslink density and, thus, the decrease in swelling rate [11]. In the end, the swelling balance is reached due to the balance between the elastic contraction force and the electrostatic repulsion force. The decrease in the swelling rate of PCA due to the adsorption of calcium ions is only 3.6%, while CA reaches 26.8%. The phenomenon means that the adsorption of PCA on calcium ions in the cement filtrate is indeed reduced. The decrease can be explained by the increased resistance and friction for calcium ions into PCA due to the smaller pore size inside PCA and the higher molecule density [17].

The ca-alginate beads also need to have a certain swelling ability to satisfy the internal curing of cement-based materials. Therefore, the diffusion behavior of water in ca-alginate beads is explored according to the swelling kinetics of ca-alginate beads in cement filtrate. The first 60% swelling data of the equilibrium swelling rate is fitted to determine the diffusion mechanism of water with the empirical formulas given by Ritger and Peppas, as follows:

(2) $$\frac{W\left(t\right)}{W_{eq}}=kt^{n}$$

(3) $$\ln\left(\frac{W\left(t\right)}{W_{eq}}\right)=n\ln t+\ln k$$

where *W_eq_* is the equilibrium swelling rate; *k* is a constant of proportionality, which is related to the network structure; *n* determines the diffusion mechanism of water molecules.

The ln (*W*(*t*)/*W_eq_*) to ln *t* curves of PCA and CA in cement filtrate are shown in Figure 6b. The relevant parameters *n* and *k* are obtained by linear fitting (shown in Table 3). The *n* values of PCA and CA in cement filtrate are both greater than 0.5, which means that the swellings of PCA and CA belong to non-Fickian swelling, and the diffusion rate of water is equivalent to the relaxation rate of ca-alginate beads. The similar *n* values indicate that the restriction of the molecular chain in the initial swelling of PCA is similar to that of CA.

The secondary kinetic model established by Schott, as shown in Equation (4), is used to determine the swelling rate and equilibrium swelling rate for the entire swelling process of PCA and CA. Equation (5) is obtained by the integral of Equation (4) according to the initial conditions *t* = 0, *W_t_* − *W*_0_ = 0, and is as follows:

(4) $$\frac{d\left(W\left(t\right)\right)}{dt}=K_{s}\left(W_{eq}-W\left(t\right)\right)$$

(5) $$\frac{t}{W\left(t\right)}=A+Bt$$

where *B* is the reciprocal of the equilibrium swelling rate (*B* = 1/W_eq_*); *A* is the reciprocal of the initial swelling rate *K*_0_ (*A* = 1/(*K*_0_ *W*_eq_^2^)).

The curves of *t*/*W*(*t*) to *t* are shown in Figure 6c. The parameters W_eq_* and *K*_0_ are obtained through linear fitting and calculation (shown in Table 3). The fitting has a high correlation. The calculated equilibrium swelling rates are consistent with the experimental results, which means that the secondary kinetic model established by Schott is suitable for evaluating the dynamic swelling process of PCA and CA. The diffusion behaviors of water in relation to PCA and CA in cement filtrate are similar. The equilibrium swelling rate of PCA is 11.11 g/g, which is significantly greater than that of CA (3.36 g/g). The higher equilibrium swelling rate is caused by lower elastic contraction force brought on by the structure of the ca-alginate beads with less chelation and higher electrostatic repulsion, caused by their higher molecular density.

Stewart proved that the diffusion behaviors of water and calcium ions are similar [17]. The swelling data of the first 60% of the equilibrium swelling rate is adopted to determine the diffusion coefficients of water in PCA and CA with Equation (6), as follows [26,27]:

(6) $$\frac{W\left(t\right)}{W_{eq}}=4{\left[\frac{Dt}{\pi D_{0}{}^{2}}\right]}^{1/2}$$

where *D* is the diffusion coefficient.

The curves of *W*(*t*)/*W_eq_* to *t*^1/2^ are shown in Figure 6d. The *D* values are obtained by linear fitting and calculation (shown in Table 3). In the cement filtrate, the diffusion coefficient of water in PCA is smaller than that of CA because the internal molecular structure of PCA is relatively denser and the pore size of PCA is smaller. The diffusion coefficient of water proves that the diffusion coefficient of calcium ions in PCA is decreased.

Although the diffusion rate of water is less than that of CA, the equilibrium swelling rate of PCA in the cement filtrate increases significantly. The equilibrium swelling rate of PCA in the cement filtrate reaches 11.11 g/g, which can satisfy the needs of the internal curing of cement-based materials.

#### 3.4.2. Swelling Kinetic in the Healing Environment

**Figure 7:**
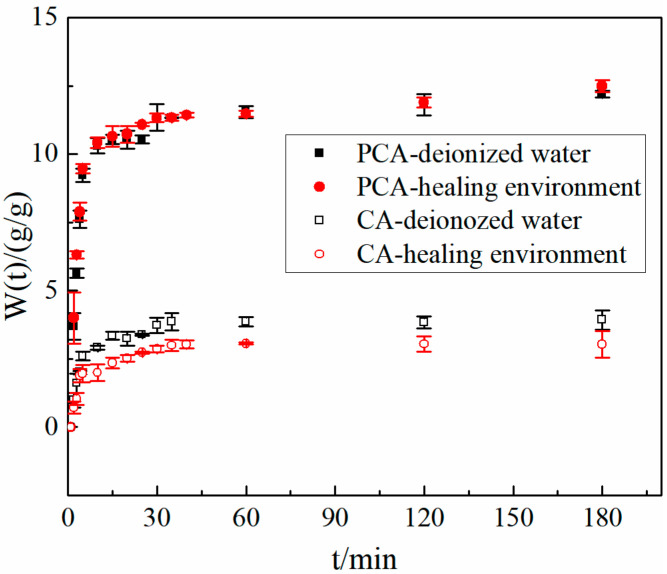
The swelling rate of two kinds of ca-alginate beads in cement filtrate over time.

Figure 7 shows the swelling behavior of PCA and CA in deionized water and the healing environment. The swelling parameters of PCA and CA in deionized water and the healing environment are shown in Table 3. The equilibrium swelling rate of PCA in the healing environment did not decrease compared with the equilibrium swelling rate in deionized water, while the equilibrium swelling rate of CA decreased by 21.9%. The decrease in the equilibrium swelling rate of CA is caused by the crosslink between CA and calcium ions in the cement filtrate.

The equilibrium swelling rate of PCA is 12.66 g/g in the healing environment, while that of CA is 3.12 g/g. This is a result of the larger molecular density of PCA and less chelation. In addition, the diffusion coefficient of water in the healing environment is still less than CA. However, the time for PCA to reach equilibrium swelling is similar to that of CA. That is probably because PCA, with its smaller size and rougher surface, has a larger specific surface area. Therefore, PCA can reach the swelling balance in the healing environment quickly, which is more conducive to the rapid closure of cracks of cement-based materials.

## 4. Conclusions

This paper proposed the mechanism and steps for preparing environmentally friendly ca-alginate beads for self-healing cement-based materials based on protonation theory. In addition, the molecular structure and the swelling rates in cement filtrate and the healing environment of ca-alginate beads prepared by the proposed method were characterized. The following conclusions can be obtained based on the above experimental results:

1. The preparation mechanism and the preparation steps of ca-alginate beads with low adsorption to calcium ions are designed based on the protonation theory. The sodium alginate powder is directly protonated and dispersed in an acid solution, where it reacts with calcium salt solution with a pH value of 7 to form ca-alginate beads in the proposed method;
2. The chelating bonds and ionic bonds have been formed in the ca-alginate beads prepared by the proposed method. The ca-alginate beads prepared by the proposed method have higher molecular density, less chelate bond content, a rougher surface, and smaller pore size than the ca-alginate beads prepared by the dropwise addition method. Furthermore, the size prepared by the proposed method is reduced by 197.74 μm;
3. The adsorption to calcium ions of the ca-alginate beads prepared by the proposed method in the cement filtrate is reduced. The diffusion coefficient of water is also reduced. However, the equilibrium swelling rate is increased due to the increased molecular content and reduced chelation, which can satisfy the need for the internal curing of cement-based materials;
4. The equilibrium swelling rate of the ca-alginate beads prepared by dropwise addition method in the healing environment is reduced by 21.9% compared with that in deionized water due to the adsorption of calcium ions. Compared with ca-alginate beads prepared by dropwise addition method, the equilibrium swelling rate of ca-alginate beads prepared by the proposed method in the healing environment does not significantly decrease compared with that in deionized water. In addition, the equilibrium swelling rate can be quickly reached due to its large specific surface area, which is more conducive to the rapid closure of cracks of cement-based materials.

## Figures and Tables

**Table 1:** The disperse phenomenon of sodium alginate powder in acid solution with different pH values.

PH Value	Phenomenon
0.01	Good dispersion, no agglomeration
0.6	Good dispersion, a small amount of agglomeration
1.42	Poor dispersion, a lot of agglomeration
2.07	No dispersersion, agglomeration
2.55	No dispersersion, agglomeration
2.95	No dispersersion, agglomeration

**Table 2:** The diameters of two kinds of ca-alginate beads.

Prepare Method	Maximum Diameter/μm	Minimum Diameter/μm	Average Diameter/μm
PCA	392.90	0.50	89.90 ± 4.61
CA	457.16	149.05	287.64 ± 6.90

**Table 3:** The swelling parameters of two kinds of ca-alginate beads in cement filtrate, deionized water, and a healing environment.

	*W_eq_* (g/g)	W_eq_* (g/g)	*K*_0_ ((g/g)/min)	*R* ^2^	*n*	*k*	*R* ^2^	*D* (cm^2^/s)	*R* ^2^
PCA-cement filtrate	11.06	11.11	0.043	0.999	0.687	0.302	0.998	2.06 × 10^−6^	0.974
PCA-deionized water	12.61	12.5	0.023	0.999	0.663	0.290	0.997	1.7 × 10^−6^	0.979
PCA-healing environment	12.50	12.66	0.032	0.999	0.599	0.325	0.996	1.95 × 10^−6^	0.993
CA-cement filtrate	3.36	3.36	0.149	0.998	0.606	0.271	0.965	1.53 × 10^−5^	0.988
CA-deionized water	3.98	4.00	0.098	0.999	0.682	0.248	0.992	1.63 × 10^−5^	0.971
CA- healing environment	3.07	3.125	0.129	0.999	0.704	0.234	0.900	6.38 × 10^−6^	0.922

## References

[1] Álvarez-Pinazo G., Cuesta A., García-Maté M., Santacruz I., Losilla E.R., De la Torre A.G., Aranda M.A.G. (2012). Rietveld quantitative phase analysis of Yeelimite-containing cements. Cem. Concr. Res..

[2] Qin L., Gao X., Su A., Li Q. (2021). Effect of carbonation curing on sulfate resistance of cement-coal gangue paste. J. Clean. Prod..

[3] Wang J.Y., De Belie N., Verstraete W. (2012). Diatomaceous earth as a protective vehicle for bacteria applied for self-healing concrete. J. Ind. Microbiol. Biotechnol..

[4] Chen Y., Gao J., Tang L., Li X. (2016). Resistance of concrete against combined attack of chloride and sulfate under drying–wetting cycles. Constr. Build. Mater..

[5] Yang Y., Lepech M.D., Yang E., Li V.C. (2009). Autogenous healing of engineered cementitious composites under wet–dry cycles. Cem. Concr. Res..

[6] Lee H.X.D., Wong H., Buenfeld N.R. (2016). Self-sealing of cracks in concrete using superabsorbent polymers. Cem. Concr. Res..

[7] Snoeck D., Schaubroeck D., Dubruel P., De Belie D. (2014). Effect of high amounts of superabsorbent polymers and additional water on the workability, microstructure and strength of mortars with a water-to-cement ratio of 0.50. Constr. Build. Mater..

[8] Jensen O.M., Hansen P.F. (2001). Water-entrained cement-based materials: I. Principles and theoretical background. Cem. Concr. Res..

[9] Jensen O.M., Hansen P.F. (2002). Water-entrained cement-based materials: II. Experimental observations. Cem. Concr. Res..

[10] Justs J., Wyrzykowski M., Bajare D., Lura P. (2015). Internal curing by superabsorbent polymers in ultra-high performance concrete. Cem. Concr. Res..

[11] Mignon A., Snoeck D., D’Halluin K., Balcaen L., Vanhaecke F., Dubruel P., Van Vlierberghe S., De Belie N. (2016). Alginate biopolymers: Counteracting the impact of superabsorbent polymers on mortar strength. Constr. Build. Mater..

[12] Engbert A., Gruber S., Plank J. (2020). The effect of alginates on the hydration of calcium aluminate cement. Carbohydr. Polym..

[13] Hu M., Guo J., Du J., Liu Z., Li P., Ren X., Feng Y. (2019). Development of Ca^2+^-based, ion-responsive superabsorbent hydrogel for cement applications: Self-healing and compressive strength. J. Colloid Interface Sci..

[14] Li X., Shen Q., Su Y., Tian F., Zhao Y., Wang D. (2009). Structure-function relationship of ca-alginate hydrogels: A novel crystal-forming engineering. Cryst. Growth Des..

[15] Ma Y., Feng Q. (2011). Alginate hydrogel-mediated crystallization of calcium carbonate. J. Solid State Chem..

[16] Liu H., Liu F., Ma Y., Douglas Goff H., Zhong F. (2020). Versatile preparation of spherically and mechanically controllable liquid-core-shell alginate-based bead through interfacial gelation. Carbohydr. Polym..

[17] Stewart T.J., Yau J.H., Allen M.M., Brabander D.J., Flynn N.T. (2009). Impacts of ca-alginate density on equilibrium and kinetics of lead (Ⅱ) sorption onto hydrogel beads. Colloid Polym. Sci..

[18] Cao Y., Shen X., Chen Y., Guo J., Chen Q., Jiang X. (2005). Ph-induced self-assembly and capsules of sodium alginate. Biomacromolecules.

[19] Ding H., Zhang L., Zhang P. (2017). Factors influencing strength of super absorbent polymer (SAP) concrete. Tianjin Univ..

[20] Chen J.P., Hong L., Wu S., Wang L. (2002). Elucidation of interactions between metal ions and ca alginate-based ion-exchange resin by spectroscopic analysis and modeling simulation. Langmuir.

[21] Dambies L., Guimon C., Yiacoumi S., Guibal E. (2001). Characterization of metal ion interactions with chitosan by X-ray photoelectron spectroscopy. Colloids Surf. A.

[22] Figueira M.M., Volesky B., Mathieu H.J. (1999). Instrumental analysis study of iron species biosorption by sargassum biomass. Environ. Sci. Technol..

[23] Moulder J.F., Chastain J., King R.C. (1992). Handbook of x-ray photoelectron spectroscopy: A reference book of standard spectra for identification and interpretation of XPS data. Chem. Phys. Lett..

[24] Snoeck D., Schroefl C., Mechtcherine V. (2018). Recommendation of RILEM TC 260-RSC: Testing sorption by superabsorbent polymers (SAP) prior to implementation in cement-based materials. Mater. Struct..

[25] Sartori C., Finch D.S., Ralph B., Gilding K. (1997). Determination of the cation content of alginate thin films by FTIR spectroscopy. Polymer.

[26] Brazel C.S., Peppas N.A. (2000). Modeling of drug release from Swellable polymers. Eur. J. Pharm. Biopharm..

[27] Okten N.S., Canakci C.C., Orakdogen N. (2019). Hertzian elasticity and triggered swelling kinetics of poly (amino ester)-based gel beads with controlled hydrophilicity and functionality: A mild and convenient synthesis via dropwise freezing into cryogenic liquid. Eur. Polym. J..

